# Extended Use of Full-Thickness Skin Grafts, Employing Variable Donor Sites

**Published:** 2018-05

**Authors:** Osman Fathy Osman, Sherif Emara

**Affiliations:** 1 Department of Plastic Surgery, Alazhar University, Cairo, Egypt;; 2 Department of Plastic Surgery, Helwan University, Cairo, Egypt

**Keywords:** Skin graft, Full-thickness, Reconstruction, Donor

## Abstract

**BACKGROUND:**

Full-thickness skin graft (FTSG) is a very versatile tool regularly used in small sizes. With the established knowledge of the graft take through the margins as well as wound bed, we extended the use of the FTSG for reconstruction of large-sized defects with satisfactory results.

**METHODS:**

We presented our experience in wound reconstruction using FTSG in 28 patients. We selected cases with graft size equal to or more than 130 cm^2^. Seven patients had chronic but healthy granulating wounds and 21 patients had fresh raw areas. Sizes of FTSG used varied between 130 to 452 cm^2^ of outstretched skin at donor sites. We used subgluteal skin crease in most of cases, though groin, upper medial thigh and medial arm aspects were also used.

**RESULTS:**

There was no difference in graft take between fresh and chronic wound sites. Almost complete graft take was the rule in all cases apart from mild epidermal skin peeling in four cases and small spots of graft necrosis in three cases. Primary wound healing at donor sites was the rule with scar hypertrophy in six cases necessitating scar conservative care for few months.

**CONCLUSION:**

Use of FTSG for reconstructing raw areas in acute and chronic wounds has to be safely reconsidered regardless of wound size. It’s still a sound and reliable tool which can decrease the necessity of complex flap coverage. Subgluteal skin crease is relatively a new donor site to be considered for large grafts with primary direct closure.

## INTRODUCTION

Skin grafting was performed in India 2000 years ago. The Scottish surgeon John Reissberg Wolfe (1824–1904) perfected the procedure of full-thickness grafting. Wolfe wrote a paper in 1875 contesting many of the accepted ideas regarding graft surgery and describing his method of reconstruction.^1^ In contemporary surgical practice, skin grafting has evolved into an essential component of skin loss management.^2^^,^^3^ Full-thickness skin grafts is an easy and versatile tool for reconstruction of skin loss whatever the cause, when it is perfectly performed it maintains a sound and reliably functioning cover to the area of use as normal skin.^3^^,^^4^

The donor site is usually hidden and can be easily closed directly. Skin at different parts of the body varies greatly in the terms of texture, color, appearance and thickness. All of these components had to be considered when choosing a donor skin area appropriate to a certain defect. Human skin varies in thickness based on its anatomical location, age, and sex of patients. Male skin is usually thicker than female skin in all anatomical locations. Children have relatively thin skin that progressively thickened until the fourth or fifth decades of life when it begins to thin once more.^3^^,^^4^

Some surgeons have bad experience with FTSG, since the graft is thick, it will need long time to heal, and it also has a higher risk of graft failure.^4^ The principal of FTSG graft revascularization through the graft margins only was completely changed. It was proven that mechanisms of FTSG revascularization through the plasmatic imbibition, capillary ingrowth and revascularization could happen at the graft margins and bed as well.^5^^,^^6^ Full-thickness skin graft is often performed for defects of the face including the nose, eye lids, and ears and hands where contraction of the graft should be minimized; it should also be used when matching the skin graft’s color to the normal skin is important.^7^

Full-thickness skin graft can also be used to reconstruct the penile urethra in one or staged procedures with satisfactory results.^8^ Tissue expansion can be also practiced to augment the amount of skin harvest at the donor site with the advantage of primary closure preserved.^8^ Full-thickness skin graft consists of the epidermis and the entire thickness of the dermis containing almost all of the skin appendages which will not only serve as a cover for raw areas, but it will also preserve multitude of the specialized skin functions.

## MATERIALS AND METHODS

In this article we presented our experience with large-sized FTSG (size more than 10×13 cm) for twenty-eight patients. Twelve patients were males and sixteen were females. Ages of patients were ranging from eight years to 74 years, and mean age was 30.8 years. The graft sizes were ranging from 13×10 cm to 25×14 cm with mean size of 18.7×11.7 cm. The patient’s clinical data are summarized in Table 1. The authors used to rely on FTSG as a reliable reconstructive tool for skin cover of raw areas at certain sites whatever the size of the area with very satisfactory results. The donor sites usually used for these extended grafts are subgluteal crease, groin, upper inner aspects of thighs, supraclavicular area, inner aspects of arms and lower abdominal crease.

**Table 1:** Patient’s clinical data

**No.**	**Age**	**Sex**	**Diagnosis**	**Recipient site**	**Donor site**	**Graft size** **(cm)**	**Result**
1	8	M	Giant hairy mole	Lower back	Subgluteal crease	19×8	Small spots of graft necrosis
2	29	F	P.B. scars	Dorsum of left hand	Left groin	18×12	Sound take
3	32	F	P.B. scars	Dorsum of left hand	Left groin	19×11	Mild epidermal peeling
4	53	M	P.B. contracture	Neck	Right groin	21×12	Sound take
5	74	F	BCC, recurrent	Temporal region	Supraclavicular	13×10	Sound take
6	12	M	P.B. contracture	Back of right knee	Subgluteal crease	18×13	Sound take
7	46	F	Skin loss, post abdominoplasty	Abdominal wall	Bilateral upper medial thigh	21×10 & 22×11	Sound take
8	41	M	P.B. contracture	Dorsum of right hand	Left groin	18×10	Sound take
9	16	F	Giant hairy mole	Face	Left groin	15×10	Sound take
10	32	M	Tattoo	forearm, Lt	Left groin	17×10	Sound take
11	18	M	P.B. contracture	Back of right knee	Subgluteal crease	19×9	Small spots of graft necrosis
12	35	F	P.B. contracture	Neck	Left groin	16×12	Sound take
13	27	F	P.B. contracture	Dorsum of right hand	Left groin	17×9	Small spots of graft necrosis
14	30	F	P.B. contracture	Dorsum of right foot	Subgluteal crease	16×9	Mild epidermal peeling
15	28	F	P.B. contracture	Back of right knee	Subgluteal crease	18×11	Sound take
16	22	F	Giant nevus	Inguinal region, left	Subgluteal crease	14×11	Sound take
17	19	M	P.B. contracture	Back of left knee	Subgluteal crease	19×12	Sound take
18	28	M	P.B. contracture	Neck	Left groin	17×10	Sound take
19	42	F	Skin loss, post abdominoplasty	Abdominal wall	Upper medial thigh	24×13	Sound take
20	36	F	P.B. contracture	Neck	Medial aspect of arm	16×9	Small spots of graft necrosis
21	39	M	Skin loss, post traumatic	Back of left leg	Subgluteal crease	15×9	Sound take
22	43	M	Skin loss, post traumatic	Loin, right	Subgluteal crease	17×14	Sound take
23	19	M	P.B. contracture	Neck & lower face	Left groin	16×12	Sound take
24	39	F	Necrotizing fasciitis	Back & sides of trunk	Both subgluteal creases	18×11 & 17×10	Sound take
25	13	M	P.B. contracture	Back of right knee	Subgluteal crease	25×14	Mild epidermal peeling
26	28	F	P.B. contracture	Dorsum of both hands	Both subgluteal creases	18×11 & 20×12	Sound take,
27	31	F	P.T. skin loss	Right Thigh	Subgluteal crease	19×12	Sound take
28	22	F	P.T. skin loss	Left loin	Subgluteal crease	18×10	Mild epidermal peeling

P.B.=Post burn; BCC=Basal cell carcinoma; P.T.=Post traumatic

From those donor sites we can harvest skin and easily close primarily without prior tissue expansion in even graft dimensions as up to 25×20 cm from the groin and subgluteal areas, and up to 16×10 cm from inner aspect of arms and supraclavicular areas. These dimensions could sometimes be increased or decreased according to the patient body mass index and degree of skin laxity. We also extensively use the post auricular and distal forearm crease but for a smaller size defect; which was not included in this study.

We usually used to meticulously circumvent the subcutaneous fat from the under surface of the graft either primarily during graft harvesting using the knife or after graft harvesting using scissors (defattening). All yellow fat must be trimmed until only the shiny white under surface of the dermis is visible; as fat is poorly vascularized and will prevent firm adherence between graft dermis and the recipient bed. Scissors for defattening have to be sharp to cut and not to crush the dermis to keep viable tissues for graft rapid revascularization. In this study we are highlighting the importance of excision of any residual white dermal remnants left at the donor site. This practice will decrease chances of hypertrophic scar formation in our experience. 

**Fig. 1:**
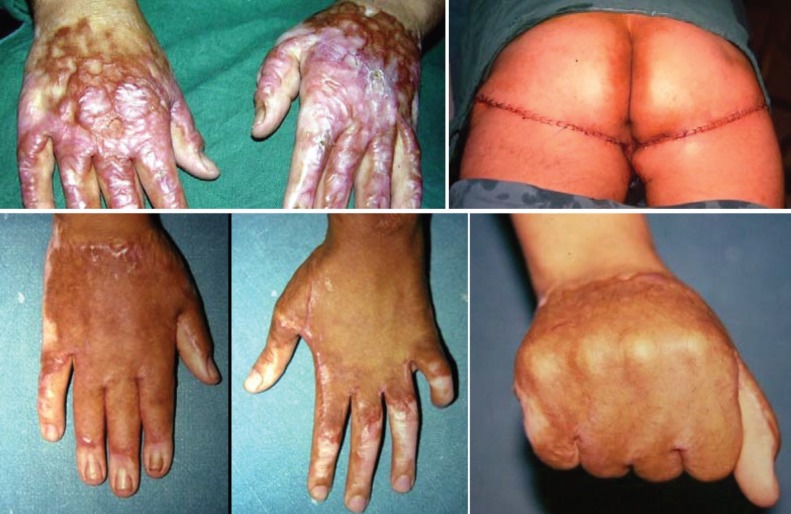
Case No. 26; 28 years old girl with post burn hypertrophic scaring in both hands. Donor sites were bilateral subgluteal creases of 18x11 cm and 20x12 cm. Graft taken well with full function regain in aesthetically accepted hands

We always plane and succeed to close the graft donor site primarily with or without limited undermining. Harvesting of subgluteal large size FTSG could be accomplished easily while the patient lying supine, through flexing the hip and knee joints, and widely exposing the subgluteal region for measurements, graft harvesting and primary closure (Figure 1). We used to remove some subcutaneous fatty tissues to facilitate wound closure exactly at the subgluteal skin crease. We usually cover the subgluteal wound dressing with an isolating polyurethane adhesive transparent film, to isolate it from perineal area as much as possible till complete healing. 

**Fig. 2:**
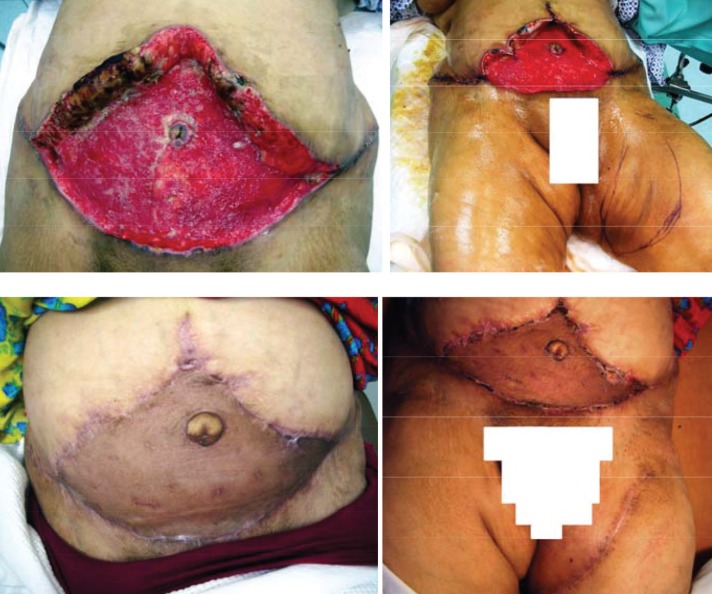
Case No. 19; 42 years old lady presented with post abdominoplasty skin loss of most of the abdominal wall. The donor site was left upper medial thigh of 13x24 cm. the graft was taken 100% with nice contour and color match compared with coverage by STSG

Full-thickness skin graft recipient site preparation has to be very meticulous. In chronic wounds the covering granulation tissue has to be healthy without debris, necrotic tissues, infections or biogenic membrane. Debridement has to continue until appearance of punctate bleeding through the healthy granulating wound surface, and without exposing bones, tendons, or joints (Figure 2). In chronic and acute wounds absolute heamostasis is mandatory as there is no way for drainage of any collection beneath the graft as compared to split-thickness skin grafts (STSG). 

The real challenge with the large size FTSG is the good graft inset and immobilization on its bed and to prevent haematoma and seroma formation. In some cases we used to fix the graft using quilting sutures between the graft and its bed to ensure good contact and prevent shearing movements with rigid splitting in certain locations. We are always used to use the tie-over sutures for dressing to fix the graft at the recipient site. A non-adherent layer such as petrolatum-based dressing is necessary to facilitate easy separation of the dressings postoperatively. 

We not only depend upon the usual absolute heamostasis at the graft bed, but also thorough washing of the graft bed with normal saline by mean of wide-pored cannula throughout the procedure and directly before dressing application, that is to wash up any possible even minor haematoma collection. The first postoperative dressing was usually done after seven days in chronic and acute raw areas. Prophylactic antibiotics were given in pre and postoperative periods according to patients and wounds flora monitoring protocol; in cooperation with the infection control department. With subsequent dressing changes, topical fucidic acid ointment was used. For donor sites, topical scar care products were usually prescribed for few months. 

## RESULTS

**Fig. 3:**
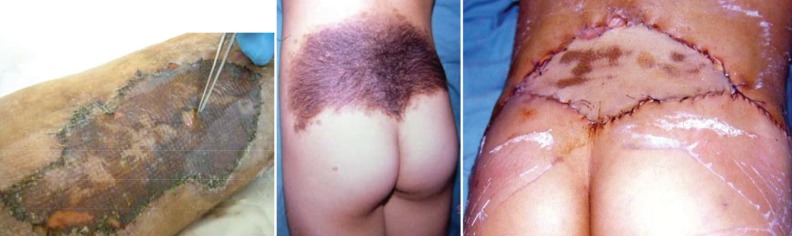
Examples of complications. Left: superficial skin peeling, Right: small patches of graft loss complicating subcutaneous collection, it was coverage of the defect (19×8 cm) resulting from excision of giant hairy mole of the middle photo (case no. 1).

Graft take was complete in all cases a part from mild epidermal peeling at small areas appeared in first dressing in four cases, which was managed conservatively with non-adherent gauze and topical antibiotic ointment on daily dressing for less than one week (Figure 3). Small spots of graft necrosis in three cases, managed through bed-side debridement and regular dressings until complete healing in less than two weeks. Surgical site infection of small area at medial end of the subgluteal donor site wounds in six patients conservatively managed, but followed few weeks after healing with scar hypertrophy, which was topically managed with satisfactory improvement.

In wide healthy granulating chronic wounds we successfully applied the FTSG, after meticulous wound debridement, with removal of all debris and securing heamostasis. Graft take was complete for all cases except in three cases in which there were small islands of graft loss due to small subcutaneous hematomas. In those three patients, bed-side wound debridement and regular dressing changes ended with epithelialization and complete healing within two weeks. There were no differences in graft take between acute and chronic wounds, provided that meticulous debridement and absolute heamostasis was done. Whatever the site and size of the grafts; early postoperative erythema or hyperpigmentation were observed in donor site scar. It was improved by time until regaining its original skin color almost within three to six months.

## DISCUSSION

In this retrospective study, we expanded the use of FTSG to almost unlimited dimensions confirming its reliability and versatility as a reconstructive tool for wound coverage; even though it is very wide. We used to present cases with raw areas up to 452 cm^2^ depending on the established facts of graft take through plasmatic imbibition, capillary ingrowth and revascularization obtained from wound margins and healthy wound beds as well.^5^^,^^6^ Through our practice, we respect variations of skin at different parts of the body varies in terms of color, texture, thickness and appearance.^9^

In reconstruction of small areas particularly at the face we have to do our best for skin matching in selecting donor sites, but this golden rule can be violated with the relatively large-sized defects. In our cases we always insist to close the donor sites directly in a linear manner. We share the opinion of many authors regarding the precautions on graft harvesting, application and fixation.^2^^-^^4^ We share the consensus of superiority of full-thickness over split-thickness skin graft, as they undergo minimal secondary contracture and maintain their robustness in less likelihood of graft trauma with more uniform texture.^9^^-^^12^

The healthy wound bed without any clinical signs of infection is an important precaution before taking the decision of grafting any raw area.^13^ This is applied to all types of skin grafting operation either split or full thickness. In our series with these large-sized defects, this precaution is amplified and dealt with extensively to decrease any possible morbidity. The importance of immobilization of the graft on its bed for the early postoperative days is stressed. We employed many techniques like tie-over dressing and, with these exceptionally large-sized grafts, we occasionally used quilting sutures for fixing the graft on its bed. This helps to fix the graft and reduce shearing forces.^11^

In some cases, we used external splints for further immobilization of the grafted areas. The hidden donor site of the subgluteal crease was preferred. This was the back-hoarse area in our study due to its great advantage to give us a large sized skin graft with minimal and most hidden donor site morbidity. The possible two disadvantages are disturbance of subgluteal sulcus in some cases with poor planning. The second is that it has a thicker skin compared to other common donor sites. Although we found that thick skin was advantageous in certain clinical scenarios. Excision of the subcutaneous fat directly beneath the subgluteal crease is suggested to facilitate primary donor site closure and to recreate the crease back. 

The use of the subgluteal donor site can provide up to 15×3 cm graft in adults with possibility of direct closure of the donor site. After limited removal of subcutaneous fatty tissues, it heals without any noticeable changes of the gluteal contour. We cannot declare that there is only limited sites of donor skin that can be closed directly as it used to be believed in harvesting FTSG. The subgluteal crease area can supply the largest hairless FTSG at a most hidden and directly closed site. In this article we introduce this relatively new donor site of FTSG with new application as FTSG is the second step in reconstructive ladder.

The aim of this article is to reintroduce a simple reconstructive surgical technique well known by all plastic surgeons, safely into a new horizon of application dimensions, with almost unlimited extents, expecting sound healing with satisfactory function and aesthetic results. The use of FTSG with these large dimensions could be of help in some clinical situations in which the alternative complex reconstructive tool, may not be easy from either the patient general or local conditions. It could also be of help in some centers with shortage of facilities for more complex flaps either local, regional, or free. 

## References

[1] Santoni-Rugiu P, Sykes P (2007). A History of Plastic Surgery.

[2] Robson MC, Krizek TJ (1973). Predicting Skin Graft Survival. J Trauma.

[3] Fitzgerald MJ, Martin F, Paletta FX (1967). Innervation of Skin Grafts. Surg Gynecol Obstet.

[4] Ratner D (1998). Skin Grafting From Here to There. Dermatol Clinic.

[5] Branham GH, Thomas JR (1990). Skin Grafts. Otolaryngol Clin North Am.

[6] Petruzzelli GJ, Johanson JT (1994). Skin Grafts. Otolaryngol Clin North Am.

[7] Pham TN, Hanley C, Palmieri T, Greenhagh DG (2001). Results of Early Excision and Full-Thickness Grafting of Deep Palm Burns in Children. J Burn Care Rehabil.

[8] Stecker JF, Horton CE, Devine CJ, McCraw JB (1981). Hypospadius Cripples. Urol Clin North Am.

[9] McGregor AD, McGregor IA (2009). Fundamental techniques of Plastic Surgery and their Surgical Applications.

[10] Chen CM, Cole J, Kryger ZB, Sisco M (2007). Skin Grafting and Skin Substitutes. Practical Plastic Surgery.

[11] Thorne CH, Thorne CH (pp). Techniques and Principles in Plastic Surgery. Grabb and Smith’s Plastic Surgery.

[12] Wani S, Matto O, Andejani D, Akmugarian F, Aldhagri F, Wafa A (2017). An innovative method of repeated tie over dressing for fixation of skin graft. World J Plast Surg.

[13] Oravcová D, Koller J (2014). Currently available skin substitutes. Cas Lek Cesk.

